# Editorial: Plant chassis for synthetic biology and its application in biomanufacturing

**DOI:** 10.3389/fpls.2024.1460378

**Published:** 2024-08-01

**Authors:** Zhu Liu, Zenglin Zhang, Sandeep Rawat, Lianyong Wang, Peijian Cao, Lei Zhao

**Affiliations:** ^1^ Key Laboratory of Engineering Biology for Low-carbon Manufacturing, Tianjin Institute of Industrial Biotechnology, Chinese Academy of Sciences, Tianjin, China; ^2^ University of Chinese Academy of Sciences, Beijing, China; ^3^ Tobacco Research Institute, Chinese Academy of Agricultural Sciences, Qingdao, China; ^4^ Govind Ballabh Pant National Institute of Himalayan Environment, Almora, India; ^5^ Department of Molecular Biology, Princeton University, Princeton, NJ, United States; ^6^ Beijing Life Science Academy, Beijing, China; ^7^ National Center of Technology Innovation for Synthetic Biology, Tianjin, China

**Keywords:** plant chassis, plant gene, synthetic biology, biomanufacturing, application

In recent years, the global food and energy crises have attracted considerable attention. Plant synthetic biology is emerging as an attractive solution to these problems, by combining plant biology with engineering principles to design and produce new devices or products that are inexpensive and easy to scale up. Plant synthetic biology uses plants as a chassis to design and construct novel biological systems with specific functions or to produce valuable compounds through techniques such as gene editing and metabolic engineering. While a significant progress has been made in plant synthetic biology in the past few years, a comprehensive understanding of the underlying biosynthetic and regulatory mechanisms remains to be explored.

This research theme contains a collection of original research papers and reviews that collectively present the latest research trends and approaches on plant chassis and plant genes in green biomanufacturing, with the aim of facilitating the wider use of plant chassis materials in biomanufacturing and the development of plant synthetic biology. Here, we highlight several studies that aim to optimize the integration of metabolic pathways and plant chassis to produce valuable compound in a cost-effective manner. Various strategies are involved, including multi-omics analysis, chassis development and gene function studies.

Tobacco, a plant chassis, has been widely used as *in vitro* culture in plant synthetic biology. Thus, the study of metabolic network in its *in vitro* culture is of great significance. This can help to promote the application of *in vitro* technology in plant propagation. To gain a comprehensive understanding of the metabolic networks in the *in vitro* culture of tobacco, Yu et al. establish a genome-scale metabolic network (GSMN), a tool designed to facilitate the characterization of overall metabolic profiles. In comparison to soil-grown tobacco, *in vitro* tobacco exhibited slower growth, reduced biomass, inhibited photosynthesis, and altered metabolites and metabolic pathways.

*Moringa oleifera* and related species has potential applications in the health, food, cosmetic, and pharmaceutical industries. Klimek-Szczykutowicz et al. present a review, summarizing the application value of *Moringa oleifera* in terms of chemical composition, nutritional properties, pharmacological activity, cosmetic applications, and agronomic importance. This review also discusses feasibility of using *Moringa oleifera* as *in vitro* culture to develop plant micropropagation techniques and for agronomic application due to its inherent bactericidal and flocculating effects. These advances provide important insights for the progressive development of strategies for the application of moringa in various fields.

Buell et al. present a new plant chassis material, *Populus*. The authors summarized the strategy for creating new morphological populus chassis materials that alter the leaf-to-wood ratio of the tree by modifying trunk branching and tree height. These morphotypes can be modified into customized types that produce high-value biofuels, bioproducts, and biomaterials not only in specific organs but also in specific cell types. Taken together, these advances demonstrate that populus can be employed as a versatile feedstock and offer new opportunities for the application of genome engineering in crop species.

The plant cuticle consists of the polyester cutin, a common cuticular waxes, which are primarily a mixture of linear very long chain fatty acids (VLCFAs) and derivatives. Alexander et al. used the synthetic biology strategy to investigate whether the maize GL2 and GL2-LIKE proteins can influence the biosynthesis of VLCFA. They found that the maize glossy2 and glossy2-like genes encode BAHD acyltransferases and they may participate in the elongation of VLCFAs. Furthermore, Liza et al. investigate the VLCFA product profile in the strain with co-expression of GLOSSY2 or GLOSSY2-like and maize fatty acid elongase component enzymes on the VLCFA product profile. The results suggest that the apparent stimulatory role of GLOSSY2 or GLOSSY2-LIKE in enabling the synthesis of longer-chain VLCFAs in diploid yeast cells may be related to the mixing of plant enzyme components with the endogenous FAE complex.

In summary, the articles in this research theme address the impact of *in vitro* culture techniques on plant chassis metabolic networks, the development of novel plant chassis materials, and plant gene function. Plant chassis research will continue to address current challenges in synthetic biology and promote its application in biomanufacturing.

## Author contributions

ZL: Conceptualization, Investigation, Writing – original draft, Writing – review & editing. ZZ: Writing – review & editing. SR: Writing – review & editing. LW: Writing – review & editing. PC: Writing – review & editing. LZ: Writing – original draft, Writing – review & editing.

